# Correction: *Factors influencing the utilisation of free-standing and alongside midwifery units in England: a qualitative research study*

**DOI:** 10.1136/bmjopen-2019-033895corr1

**Published:** 2020-03-13

Walsh D, Spiby H, McCourt C, *et al*. Factors influencing the utilisation of free-standing and alongside midwifery units in England: a qualitative research study. *BMJ Open* 2020;10:e033895. doi: 10.1136/bmjopen-2019-033895.

This article has been corrected since it was published. The affiliations for Christine McCourt and Lorraine Culley are updated. Also, the NICE Intrapartum Care guideline in ‘INTRODUCTION’ and ‘CONCLUSIONS’ sections is corrected as CG190.

