# Supplementary material for: Factors influencing the utilisation of free-standing and alongside midwifery units in England: a qualitative research study
Source: BMJ Open. 2020 Feb 17;10(2):e033895. doi: 10.1136/bmjopen-2019-033895 (PMC7045002; doi:10.1136/bmjopen-2019-033895)
Supplement: Author's manuscript [file bmjopen-2019-033895.draft_revisions.pdf]

BMJ Open is committed to open peer review. As part of this commitment we make the peer review history of every article we publish publicly available.

When an article is published we post the peer reviewers' comments and the authors' responses online. We also post the versions of the paper that were used during peer review. These are the versions that the peer review comments apply to.

The versions of the paper that follow are the versions that were submitted during the peer review process. They are not the versions of record or the final published versions. They should not be cited or distributed as the published version of this manuscript.

BMJ Open is an open access journal and the full, final, typeset and author-corrected version of record of the manuscript is available on our site with no access controls, subscription charges or pay-per-view fees (http://bmjopen.bmj.com).

If you have any questions on BMJ Open's open peer review process please email info.bmjopen@bmj.com

BMJ Open

Factors influencing the utilisation of free-standing and alongside midwifery units in England: A Mixed Methods Research Study

Journal:	*BMJ Open*
Manuscript ID	bmjopen-2019-033895
Article Type:	Original research
Date Submitted by the Author:	28-Aug-2019

Complete List of Authors:	Walsh, Denis; University of Nottingham, School of Health Sciences<br>Spiby, Helen; University of Nottingham Faculty of Medicine and Health Sciences<br>McCourt, Christine; City University<br>Grigg, Celia<br>Coleby, Dawn; Coventry University<br>Bishop, Simon; University of Nottingham<br>Scanlon, Miranda; City University<br>Culley, Lorraine; De Montfort University<br>Wilkinson, Jane<br>Pacanowski, Lynne<br>Thornton, Jim; University of Nottingham

Keywords:	OBSTETRICS, Organisation of health services < HEALTH SERVICES ADMINISTRATION & MANAGEMENT, Health policy < HEALTH SERVICES ADMINISTRATION & MANAGEMENT

SCHOLARONE™
Manuscripts

I, the Submitting Author has the right to grant and does grant on behalf of all authors of the Work (as defined in the below author licence), an exclusive licence and/or a non-exclusive licence for contributions from authors who are: i) UK Crown employees; ii) where BMJ has agreed a CC-BY licence shall apply, and/or iii) in accordance with the terms applicable for US Federal Government officers or employees acting as part of their official duties; on a worldwide, perpetual, irrevocable, royalty-free basis to BMJ Publishing Group Ltd ("BMJ") its licensees and where the relevant Journal is co-owned by BMJ to the co-owners of the Journal, to publish the Work in this journal and any other BMJ products and to exploit all rights, as set out in our licence.

The Submitting Author accepts and understands that any supply made under these terms is made by BMJ to the Submitting Author unless you are acting as an employee on behalf of your employer or a postgraduate student of an affiliated institution which is paying any applicable article publishing charge ("APC") for Open Access articles. Where the Submitting Author wishes to make the Work available on an Open Access basis (and intends to pay the relevant APC), the terms of reuse of such Open Access shall be governed by a Creative Commons licence – details of these licences and which Creative Commons licence will apply to this Work are set out in our licence referred to above.

Other than as permitted in any relevant BMJ Author's Self Archiving Policies, I confirm this Work has not been accepted for publication elsewhere, is not being considered for publication elsewhere and does not duplicate material already published. I confirm all authors consent to publication of this Work and authorise the granting of this licence.

For peer review only

Title Page

Title:

Factors influencing the utilisation of free-standing and alongside midwifery units in England: A Mixed Methods Research Study

Authors:

Dr Denis Walsh, Retired Associate Professor (Lead and Corresponding Author)

University of Nottingham

Nottingham

UK

Postal Address:

116 Letchworth Rd, Leicester LE3 6FH

Telephone: 07905735777

Email: Denis.walsh@ntlworld.com

Helen Spiby, Co-Author, Professor in Midwifery

University of Nottingham

Nottingham

UK

Christine McCourt, Co-Author, Professor in Maternal Health

City University

London

UK

Dr Celia Grigg, Co-Author, Research Fellow

University of Auckland

Auckland

New Zealand

Dr Dawn Coleby, Co-Author, Senior Research Fellow

For peer review only

Coventry University

Coventry

UK

Dr Simon Bishop Co-Author, Senior Lecturer in Business School

University of Nottingham

Nottingham

UK

Dr Miranda Scanlon Co-Author, Service User

City University

London

UK

Lorraine Culley Co-Author, Emeritus Professor of Social Science & Health

De Montfort University

Leicester

UK

Jane Wilkinson, Co-Author, GP Commissioner

Wirral Community NHS Foundation Trust

Liverpool

UK

Lynne Pacanowski, Co-Author, Retired Head of Midwifery

Guys & St Thomas' NHS Trust

London

UK

Jim Thornton, Co-Author, Professor of Obstetrics

University of Nottingham

Nottingham

For peer review only

UK

Key Words: Obstetrics, Qualitative Research, Midwifery Units

Word Count: 4975

For peer review only

ABSTRACT: Factors influencing the utilisation of free-standing and alongside midwifery units in England: A Mixed Methods Research Study

Objective: To identify factors influencing the provision, utilisation and sustainability of midwifery units (MUs) in England

Design: Case studies, using individual interviews and focus groups, in six NHS Trust maternity services in England

Setting: NHS maternity services in different geographical areas of England

Participants: Maternity care staff and service users from 6 sites: 2 sites where more than 20% of all women gave birth in MUs, 2 sites where less than 10% of all women gave birth in MUs and 2 sites without MUs. Within each site, individual interviews were done with clinicians, managers, commissioners, and 4 focus groups were conducted, 2 with midwives and 2 with service users.

Interventions: Establishing MUs

Main Outcome Measures: Factors influencing MU use

Results: The study findings indicate most Trust managers and clinicians do not regard their MU provision as being as important as their obstetric unit (OU) provision and therefore it does not get embedded as an equal and parallel component in the Trust's overall maternity package of care. The analysis illuminates how implementation of complex interventions in health services is influenced by a complex range of factors including the medicalisation of childbirth, perceived financial constraints and institutional norms protecting the status quo.

Conclusions: There are significant obstacles to MUs reaching their full potential, especially free-standing midwifery units (FMUs). These include the lack of commitment by providers to embed MUs as an essential service provision alongside their OUs, an absence of leadership to drive through these changes and the capacity and willingness of providers to address women's information needs. If these remain unaddressed, childbearing women's access to MUs will continue to be restricted.

Strengths and limitations of this study

1. The richness and breadth of data captured across multiple case study sites with contrasting organisational characteristics
2. The focus groups generated discussion and insight unlikely to be obtained by individual interviews and were particularly effective in comparing service user perspectives with provider perspectives from within the same case.
3. The CFIR framework utilised for analysing the case study data was helpful in organising data collection and analysis to identify factors that impacted on implementation.
4. We were unable to get access to Trust documentation regarding MU policies and organisation which may have helped triangulate data from the interviews and focus groups.
5. We were only able to recruit one finance director, and this may have reduced the comprehensiveness of our finance related data.

Funding

For peer review only

This work was supported by the National Institute for Health Research Health Services and Delivery Research programme (Ref: 14/04/28).

Competing Interest Statement

Professor Thornton reports being a member of the HTA and EME Boards. Dr Scanlon reports grants from NIHR, during the conduct of the study; personal fees from "WHICH?", grants from NIHR, personal fees from National Perinatal Epidemiology Unit, personal fees from Rod Gibson Associates Ltd, personal fees from Midwifery Unit Network, outside the submitted work. No other completing interests have been declared.

For peer review only

INTRODUCTION

Since 1993, maternity care policy in England has promoted women's choice of place of birth. This became the national choice guarantee in Maternity Matters in 2007[1] which stipulated women should have three options: birth in a maternity hospital (obstetric unit or OU), birth in two types of midwifery unit (MU) either alongside (AMU) or freestanding (FMU) or birth at home. AMUs are attached to existing maternity hospitals while FMUs are geographically separate. The Birthplace in England cohort study[2] reported that outcomes for low risk pregnant women were better and costs reduced if birth occurred in MUs, both AMUs and FMUs, rather than OUs. For example, having a baby in a MU reduced caesarean section rates by two thirds, while there was no difference in adverse neonatal outcomes.

The most recent NICE guidelines on intrapartum care therefore recommend MUs for low risk women, i.e. women without significant health risk factors who are predicted to have a normal labour and birth[3]. Sandall and colleagues' research suggests this could be around 45% of all birthing women[4]. However, despite the advantages of MUs, the National Audit Office (NAO) found that only 11% of women gave birth in those settings while the vast majority continue to give birth in OUs[5]. In addition, MUs were not equally distributed across the country[5]. A third of NHS Trusts had no MUs, and those that did, were frequently underutilised with less than 10% of all births occurring in them. If 20% of births occurred in MUs, savings to the NHS maternity budget could be around £85 million/year, projecting from average cost differences[6]. This represents a 3% saving on the current annual budget of £2.6 billion for maternity care[7].

These changes in guidelines and a policy emphasis on patient or consumer choice are in line with the direction of national policy across wider areas of healthcare. Midwifery units could be considered an example of a complex health service 'intervention' that is a change in organisation models, based on best clinical evidence, that require a systemic, multi-stakeholder approach to implementation. A range of prior studies have highlighted challenges in the implementation of health policies and evidence of this nature[8-10].

There has been no specific research investigating the reasons for the highly varied rates of MU provision across England. To rectify this, an NIHR-funded research project was conducted to explore the reasons for these anomalies in the provision of MUs in England. This novel, mixed-methods exploration of maternity service provision was delivered by a multidisciplinary team and supported by a service-user reference panel. The principal objectives of the study were to describe the configuration, organisation and operation of MUs in England and identify key barriers to the uptake of MU care. A three-phase mixed methods study incorporating a Mapping Survey, Comparative Case Studies and a Stakeholder Workshop was undertaken to answer these objectives. The national mapping of MUs and OUs nationally (including numbers and organisation) has already been reported[11].

The most significant finding of the mapping phase (which included all 134 NHS Trusts providing maternity services) was that, although the percentage of births in MUs has increased from 5% to 14% since the Birthplace in England study, that growth has occurred in AMUs. This falls well short of the potential percentage of births in MUs of 36%, extrapolated from Sandall's study[4]. The mapping phase also identified organisational processes within maternity services regarding MU access and utilisation. Two key findings were, firstly that 97% percent of AMU midwives and 50% of FMU midwives were moved regularly during shifts, usually to the OU. Staff shortage or 'capacity issues' were the primary reason given

For peer review only

for MU closures, which occurred for 28% of AMUs and 39% of FMUs. Thus, some MU midwives were providing care for low risk women in OUs while AMUs and FMUs stood vacant. AMUs that were underutilised (i.e. <10% of births) were closed three times as frequently as AMUs where >20% of women gave birth. Secondly, AMU admission rates were facilitated in some settings by maternity services operating an opt-out policy i.e. women who met eligibility criteria were defaulted to them unless they opted otherwise, rather than a more traditional OU opt-out policy. Of the high-performing Trusts with AMUs, 73% had an opt-out policy compared with only 14% of the low-performing Trusts with AMUs.

Here we report the methods and findings for phase 2 of our overall study, to explore the factors influencing the utilisation of MUs and to understand in more depth the picture obtained in the mapping survey. The study findings address the specific challenges for maternity care but also illuminate wider issues relevant to implementation science in health.

METHODS

We conducted qualitative case studies to understand and compare maternity services with different levels of progress in implementation. Based on our mapping survey findings, we chose six case-study sites (NHS Trust maternity services including all units) to study in-depth. Two were high-performing (our definition: MUs achieving 20% or more of all local facility-based births), two were low-performing (MUs achieving 10% or less of all local births) and two sites had no MUs. Data collection from each site involved: individual interviews with senior managerial and clinical midwives, obstetricians and neonatologists, Trust CEOs and commissioners in each case study site (n=57); 13 focus groups with clinical midwives (n=60); 13 focus groups with women who had recently used maternity services (n=52). All focus groups and interviews were recorded, professionally transcribed and then analysed thematically, with both a 'within-case' and 'cross-case' comparison. This was assisted by use of the Consolidated Framework for Implementation Research (CFIR) which provides a list of constructs previously found to impact on the process of implementing evidence at an organisational level across healthcare organisations[12]. Constructs are presented within the CFIR under five domains: 1) the 'outer' wider health system (policies) and society (norms), 2) the characteristics of the individuals involved (beliefs, preferences), 3) the 'inner' context of the relevant organisations i.e. NHS Trusts – their culture, networks etc, 4) the context and nature of the 'intervention' – in this case MUs and 5) the process of change (implementing the intervention).

Data analysis involved coding qualitative data, initially using open coding to identify potential themes and then mapping these to the constructs of the CFIR, first on a 'within case' and then on a comparative cross-case basis, with cross case analysis guided by the question of why some services were successful in opening, utilising and sustaining MUs and others were not.

Following analysis, we convened a workshop of 56 stakeholders from across England comprising provider, commissioner, education and service user constituencies for phase three. Findings were presented, and focused discussion groups identified a set of priority actions to help services to increase the provision and uptake of MUs.

Ethical approval was granted for phase 2 of the study the West Midlands - Solihull Research Ethics Committee (IRAS ID 200356) as phase 1 and 3 were deemed service development.

For peer review only

Participant consent was obtained for involvement in all interviews and focus groups.

Public and Patient Involvement

Public involvement was integrated into the study throughout all phases including project design, implementation, management and dissemination. One of the Co-Investigators was a service user and contributed to the original idea for the research and to developing the research protocol. Four service users were recruited to a service user reference group from an established local service user maternity network. This group reviewed all aspects of the study design, including the study documents. Group members advised on approaches to achieve recruitment of women into focus groups and participated in facilitating the women's focus groups at the six case study sites. They also co-presented the preliminary findings at the Stakeholder Workshop and co-facilitated two of the small group discussions at this event. They will also be involved in the dissemination of findings via their Facebook groups.

Additional aspects of the methods, more detail on the analytical approach across all three phases, reflections on the utility of the CFIR framework, sample sizes and composition will be available electronically in the Final NHIR Report.

RESULTS

The case study analysis distilled key themes that need addressing if English maternity services are to maximise the clinical, psychosocial, workforce and economic benefits of MUs. These will be reported under the various domains of the CFIR framework.

Outer Setting

We found strong institutional and societal pressure (risk and litigation policies, fiscal constraints) to maintain OUs as the core focus of maternity care, positioning MUs as a lesser priority and an optional extra. This involved a number of elements, including legal and governance frameworks, professional hierarchies and resource flows, which contributed to the dominance of OU care. Particularly important were perceptions of appropriate approaches to managing risk, present in the responses of representatives from all professional groups:

"There's also the potential clinical risks of people giving birth in those areas (AMUs). And we had an unfortunate death about three years ago.." [Obstetrician]

"There might be a degree of fear that if people started saying that, you can go in there (to the MU), you are constantly reminded that women have to be told the risks. ...because of the litigation now." [Midwife in focus group]

Another factor to emerge from interviews, especially from service providers, was budget constraints. Financial cutbacks within Trusts were mentioned across all sites as frustrating the development of MUs:

"I think the whole financial situation within the Trust at the moment is a driver. ... Unfortunately, all our finance team will only see is the figure at the bottom of the page. ...it is a sort of finance driven organisation and you're forever trying to find ways of saving money, cutting costs, etc" [Midwifery Manager]

All respondents appeared to accept the need for Trusts to save money as a 'fait accompli' and the unaffordability of MUs as a 'fact' as typified by the phrase '*we're in a period of austerity now*' from one interviewee and positioned maternity as competing and losing out to other services.

Overall, factors in the 'outer setting' of midwifery could be seen as contributing to a 'medical' view of childbirth that shaped perceptions of where birth should be situated. This was highlighted in women's focus groups:

"..we've been become accustomed to birth taking place in hospital (OUs) and to step outside that model you've got to face your family and peers and actually have a good reason why you want to birth outside that accepted model…hospital is perceived as safest, the 'just in case' option.."

Characteristics of Individuals

Closely related to a medicalised view of childbirth, we found mixed beliefs among individuals about the efficacy of MUs, with pockets of strong scepticism across high and low uptake sites. In many instances, these attitudes 'drowned out' opposing views emanating from the extant evidence. Antipathy towards MUs was particularly strong in the case of FMUs, in relation to which several common assumptions were noted. These included the perceived superior safety of the medical model, that FMUs and AMUs offer essentially an identical service and that FMUs are not popular with women:

I think majority of women and all my friends will opt for an alongside MU, because most women do want the option of midwifery led but if anything goes wrong they just want to go down that corridor, through that door. [Midwifery Manager]

Many midwives, especially in sites with no MUs, were reported as actively resisting the development of an FMU:

"..they (the midwives) were completely horrified at the idea of having a standalone midwifery-led unit" [Midwifery Manager]

While variations of this attitude could be found across all sites, within high performing sites we did find existing AMU and FMU 'champions' who saw themselves as contributing to the 'mission' or 'vision' of midwifery led birth:

"*The vision is to up the numbers, so we have the quality boards, and we are aiming to increase the deliveries in the midwifery led unit, and home deliveries. …we are continuously striving to increase it."* [Midwife]

Inner Setting

We found that collaboration between MUs and OUs was important for the successful embedding of the MU model, and pockets of excellent collaborative relations were reported within high performing sites. More commonly, this did not occur, creating an 'us and them' atmosphere as illustrated by this segment of a focus group transcript between an FMU midwife and the facilitator:

Int: *I went on a transfer, and the reception I got was non-existent.*
Fac: *What do you mean?*
Int: *There was nobody waiting…there wasn't a cot in the room, no midwife came, I had to find somebody.*
Fac: *But they're always told ahead that you're coming?*
Int: *Oh yeah, they know you're coming. I've been greeted with oh, here comes another failure from FMU.*

We also found evidence in some Trusts of a culture of marginalising and undervaluing of FMUs. As a result, several FMUs were under threat of closure, even in high-performing sites. The two dominant rationales for closure were that they are under-used and too expensive as illustrated by these quotes:

"Well it (FMU) is small and we do have to understand how viable it (FMU) is because you can't spread yourself so thin. So it (FMU) is difficult to manage because we're covering so many other areas, and the birth rates numbers are very low" [Manager]

"If you spoke to any of the consultants I am sure they would say it [FMU] should be closed because it's a waste of money. And it's an unfair allocation of resources, in a relatively resource poor environment." [Manager]

In addition, we found evidence that FMUs can be subjected to a mixture of managerial neglect and authoritarian control from their host Trusts. An FMU manager said:

"They (Trust management) always pay us lip service… don't promote us'…we've been fighting for a year to get a video on the Trust website, of a tour of our birth centre…. You do feel like the poor relation". [Focus Group Midwife]

This manifested in several contradictory messages coming from some Trusts. We found examples of all of the following: using FMUs to solve capacity crises at times of peak activity while threatening them with closure at other times; restricting opportunities for FMU staff to promote their services as illustrated in the quote above; FMU midwives experiencing a negative reception in OUs when transferring women in labour; FMU staff not being consulted on strategic changes that impacted on them.

Intervention Characteristics

Although clearly recommended by evidence, embedding MU provision was perceived as presenting a number of challenges. MUs are intended to provide care to low risk women, where midwives can practice the skills of normal midwifery. However, a number of midwife respondents felt that practicing within them required different skills and a level of confidence, which they were not well prepared for.

"Because everyone has worked in such a high-risk environment, you become deskilled to an extent, and feel a bit apprehensive about normal birth… you know, trusting that women can have babies low risk." [Focus Group Midwife]

Midwifery managers and midwives in our study recommended mandatory training in normal birth skills to address this concern. Linked to a perceived deficit in skills and arguably more

For peer review only

influential was a lack of confidence amongst midwives to make decisions in MU settings where midwives are more autonomous, as illustrated from this midwife focus group:

"One of the effects that that has had, is that it has decreased a lot of the midwives' confidence in this unit, of providing low risk care, because they don't have the environment, a consistent one, in which to do it properly…. when you're on labour ward you become over reliant on the doctors.."

Process

There were considerable differences across sites in the process of implementing and developing MUs. Leadership emerged as key to successful implementation.

"it's crucial to have an inspirational leader. If you don't have somebody at the very top who is passionate about it (MUs) happening, it won't happen. And they must cascade, get everybody onboard." [Midwives Focus Group]

"a charismatic leader to kind of bring it together…unless you've got that then I think it's quite hard to bring it to fruition." [Manager]

Continuity of leadership contributing to organisation memory was also stressed:

"I think the birth centres are being used less at the moment, and that does seem to coincide with a change of leadership." [Midwives Focus Group]

Only a few sites had an active policy of the ongoing promotion of MUs to their local women to increase and sustain their utilisation:

"So you have to do a lot of positive promotion, you have to get out there. And you're almost selling a product. And that's how we saw it. So we did lots of promotional events, and got lots in the press, about the opening of the FMU." [Manager]

Successful implementation was also dependent on a clear clinical pathway from the beginning of pregnancy until the onset of labour.

There was a wide variation in the information women had, or were given, about MUs - within and between Trusts. The majority of women in the focus groups reported not receiving information. Participants from five of the six case study Trusts mentioned not being given information about the local MUs (including the two which have more than 20% of women giving birth in a MU).

"Well it's just that nobody gave us the information about it [MUs]. That's the main thing. I didn't know nothing about it. I didn't even know it even existed." [Women's Focus Group]

Women expressed concerns about the place of birth booking process, such as whether it was necessary to decide at the beginning of pregnancy, how it was done, and if it was possible to change your mind.

For peer review only

"I wasn't aware that you had to decide before you went in for your booking appointment, so I was asked on the spot and I didn't know. But the midwife said that you have to choose now because they have to book the hospital in advance." [Women's Focus Group]

DISCUSSION

This research has illuminated why MUs are underused in England and still not available in many NHS Trusts. The central challenge in all case study sites was introducing and sustaining what was still perceived as an alternative configuration (MUs) into an existing and embedded mainstream, 'taken for granted' model (OUs) which has been in place for the past five decades. OUs are the default place of birth for the vast majority of women in England, regardless of women's risk profiles.

Several external (outer context), and internal (inner context) factors combine to reinforce the status quo, and these are augmented by personal beliefs of key players, intrinsic features of the service, and the process of change adopted to implement the intervention. Coxon[13 14] and Scamell[15] argue that the construction of birth as risky in policy initiatives and by service providers over recent decades has shaped women's preference for birth in OUs. Birth as a risky endeavour is a by-product of the medicalisation of childbirth over a similar period that has seen caesarean section rates rise exponentially[16 17]. As Coxon demonstrated, if women's first experience of birth is in a hospital labour ward, they are likely to choose the same for subsequent births[13].

Financial constraints within Trusts were often seen as limiting the development of MUs. While economic evaluations suggest the overall economic outcomes of increasing births in MUs is positive[18], the start-up costs were seen as a barrier, and the longer-term savings from lower morbidity in the target population that accrue across the health system were not recognised. In a climate of scarcity, new ways of structuring care must demonstrably save money, or at least, be perceived to, in the short term.

Despite national guidelines based on extensive evidence recommending MUs for women at low obstetric risk, we found that managers and clinicians in provider settings harboured considerable ambivalence about the safety of MUs, even among midwives. Research has shown that personal belief can moderate evidence[19] and is a key variable to address in systematic reviews of what facilitates the translation of evidence into practice[20 21]. FMUs were especially vulnerable to negative beliefs about their efficacy even though they pre-date AMUs by decades, albeit under the title of maternity homes or GP units. Though AMUs are a relatively new phenomenon, there has been an exponential increase in their use over the past 6 years, even if still at a low overall level, which could reflect the broader bias favouring the embedded system of OUs as AMUs are co-located with them.

A defining characteristic of MUs as an intervention is that their functioning is entirely dependent on midwives because they are midwife-led and managed. Skills in managing normal labour and birth and decision-making autonomy are prerequisites for practise in this setting. Our findings highlighted both a lack of midwifery confidence and skill that can be traced back to the training and practice of UK midwives within predominantly obstetric-led services. Numerous surveys and papers have demonstrated this over the last 30 years since Robinson's pioneering research on the loss of traditional midwifery skills in the 1980s[22-25].

For peer review only

Our findings pinpoint the importance of leadership to the process of managing organisational change of this magnitude. Best et al's realist review[26] found that blending designated leadership (someone in charge of the programme) with distributed leadership (professionals/partner organizations sharing responsibility for delivering it) was the most successful at embedding and sustaining change. For the successful development and operationalisation of MUs, leadership needs to be exercised vertically via the layers of organisational hierarchy and horizontally across different professional groups; and at each of these levels, designated leadership and distributive leadership should be combined. An important component of leadership was the identification and subsequent impact of having 'champions'. Champions of MUs were either clinicians, managers or service users who were highly influential in promoting the service and recruiting support for it.

Designated leaders working with champions were better at establishing clinical pathways for women to optimise access and utilisation of MUs. This included user friendly information to promote the choice of MUs, adopting an opt-out mechanism for AMUs and employing a consultant midwife to oversee and develop MUs.

This study contributes to understanding of the use of CFIR framework for understanding complex interventions which requires major organisational change in their delivery. In this case the 'new' service also required close partnership with a historically embedded model, with the intention that care delivery is more equally shared across both. The domains and constructs of the CFIR framework usefully provided a heuristic to discuss and analyse individual factors playing into the challenges of implementation. However, it provided less of a template to consider the complex interaction of factors which cut across organisational boundaries and levels of analysis which, for example, entangled the 'inner' and 'outer' setting. Issues such as perverse incentives to prioritise short term cost savings can be seen as present within the wider policy environment but are also constitutive of relations between organisations and actors at the meso and micro level. Similarly, the 'process' domain of the framework assumes a discernible and comparable period of implementation; in our study we found the process of 'implementation' in fact occurring over the long term, with ruptures, discontinuities and cycles of growth and contraction in use of the 'new' services. More recently published frameworks have sought to take greater account of wider contextual factors[27] and these could be considered for future implementation studies of complex interventions.

The strength of this comparative case study methodology is the richness and breadth of data captured across multiple sites with differing organisational characteristics. In addition, focus groups generated discussion and insight unlikely to be obtained by individual interviews. They were particularly effective in comparing service user perspectives with provider perspectives from within the same case.

Inevitably, we were not able to include a full range of service users in the focus groups as we did not have translation services. Despite this, we did have BAME (Black, Asian and Minority Ethic) representation in some of the focus groups. Despite considerable efforts, we were only able to recruit one Finance Director, and this may have reduced the comprehensiveness of finance related perspectives.

IMPLICATIONS

For peer review only

The key implication of this research is that, in many areas of England, women at low risk of complications are being denied the maternity care that evidence shows is most suitable for them, because of the factors highlighted in this paper.

The importance of leadership was a principal finding from our cases studies as it is a critical factor in the normalisation of an intervention to the point where it is no longer appraised as marginal but becomes incorporated and understood as a core part of the service[28].

It was clear from our study that inequality of access to information is primarily a matter of women not being given information about the option of MU care. Having an opt-out policy for FMUs should also be explored. FMUs have the additional advantage of being a more local provision for some women and therefore meeting the wider health service principle of moving care closer to home[29]. In addition it is clear that women need a higher quality of information about place of birth options and evidence, which should be provided at different stages of the pregnancy[30].

Addressing the plight of FMUs is urgent in the current climate. Trusts need to value their FMU(s) as central to the broader maternity service provision and an important choice for low risk women. In particular, the common perception that FMUs are a financial burden unless operating at maximum capacity needs to be challenged as the available evidence suggests they are cheaper than birthing the same women in an OU, even when they are operating at around 30% capacity. This is because health economists factored in the savings they generate in reduced intervention and maternal morbidity[6 18]. FMU facilities could also be used more extensively for other outpatient services and could arguably operate as part of a community hub as envisioned by the Implementing Better Births policy document[31 32].

Finally, though the CFIR framework utilised for this research was helpful in making explicit generic categories that impact on organisational change within our case study sites, it was less useful when complex interventions have been introduced over varying time periods as some of the domains of the framework e.g. process, assume a comparable timeline. This should be noted by other researchers intending to use implementation science approaches.

CONCLUSIONS

Nearly five years on from NICE Intrapartum Care Guidance recommending birth in MUs for low risk women, MUs across England are not fulfilling their potential. This is because of the challenge of embedding them into the existing hospital-based OU model, that has been in place for several decades. Changing the status quo requires leadership from both commissioners and providers and a clearly articulated belief in the value of MUs, exercised through committing resources, streamlining care pathways and ongoing promotion to service users.

REFERENCES

1. Health Do. Maternity Matters: Choice, access and continuity of care in a safe service: NHS, 2007:56.

For peer review only

2. Brocklehurst P, Hardy P, Hollowell J, et al. Perinatal and maternal outcomes by planned place of birth for healthy women with low risk pregnancies: the Birthplace in England national prospective cohort study. *British Medical Journal* 2011;343(7840):d7400.
3. NICE NIoHE. Intrapartum Care: Care of healthy women and their babies during childbirth. Clinical Guideline 190 Methods, evidence and recommendations. 2 ed. London: NICE, 2014.
4. Sandall J, Murrells T, Dodwell M, et al. The efficient use of the maternity workforce and the implications for safety and quality in maternity care: a population-based, cross-sectional study. *Health Serv Deliv Res* 2014;2(38) doi: 10.3310/hsdr02380
5. NAO. National Audit Office (2013) Maternity services in England. London: NAO. London, 2013.
6. Schroeder E, Petrou S, Patel N, et al. Cost effectiveness of alternative planned places of birth in woman at low risk of complications: evidence from the Birthplace in England national prospective cohort study. *BMJ: British Medical Journal* 2012;344
7. Walsh D, Spiby H, McCourt C, Bishop S, Dodwell M, Culley L, Pacanowski L, Wilkinson J, Thornton J. HS & DR: NIHR, 2014:122.
8. Greenhalgh T, Papoutsi C. Spreading and scaling up innovation and improvement. *BMJ* 2019;365:l2068.
9. May C, Finch T, Mair F, et al. Understanding the implementation of complex interventions in health care: the normalization process model. *BMC Health Services Research* 2007;7(1):148.
10. Hasson H. Systematic evaluation of implementation fidelity of complex interventions in health and social care. *Implementation Science* 2010;5(1):67.
11. Walsh D, Spiby H, Grigg CP, et al. Mapping midwifery and obstetric units in England. *Midwifery* 2018;56(Supplement C):9-16. doi: https://doi.org/10.1016/j.midw.2017.09.009
12. Damschroder LJ, Hagedorn HJ. A guiding framework and approach for implementation research in substance use disorders treatment. *Psychology of Addictive Behaviors* 2011;25(2):194-205. doi: 10.1037/a0022284
13. Coxon K, Chisholm A, Malouf R, et al. What influences birth place preferences, choices and decision-making amongst healthy women with straightforward pregnancies in the UK? A qualitative evidence synthesis using a ‘best fit’framework approach. *BMC pregnancy and childbirth* 2017;17(1):103.
14. Coxon K, Sandall J, Fulop NJ. To what extent are women free to choose where to give birth? How discourses of risk, blame and responsibility influence birth place decisions. *Health, risk & society* 2014;16(1):51-67.
15. Scamell M. Childbirth Within the Risk Society. *Sociology Compass* 2014;8(7):917-28. doi: 10.1111/soc4.12077
16. Johanson R, Newburn M, Macfarlane A. Has the medicalisation of childbirth gone too far? *BMJ: British Medical Journal* 2002;324(7342):892.
17. MacKenzie Bryers H, van Teijlingen E. Risk, theory, social and medical models: a critical analysis of the concept of risk in maternity care. *Midwifery* 2010;26(5):488.
18. Schroeder L, Patel N, Keeler M, et al. The economic costs of intrapartum care in Tower Hamlets: A comparison between the cost of birth in a freestanding midwifery unit and hospital for women at low risk of obstetric complications. *Midwifery* 2017;45:28-35. doi: http://dx.doi.org/10.1016/j.midw.2016.11.006
19. Roome S, Hartz D, Tracy S, et al. Why such differing stances? A review of position statements on home birth from professional colleges. *BJOG: An International Journal of Obstetrics & Gynaecology* 2016;123(3):376-82.
20. Ivers N, Jamtvedt G, Flottorp S, et al. Audit and feedback: effects on professional practice and healthcare outcomes. *Cochrane Db Syst Rev* 2012(6) doi: 10.1002/14651858.CD000259.pub3
21. Forsetlund L, Bjørndal A, Rashidian A, et al. Continuing education meetings and workshops: effects on professional practice and health care outcomes. *Cochrane Db Syst Rev* 2009(2) doi: 10.1002/14651858.CD003030.pub2

For peer review only

22. Robinson S. Normal maternity care: whose responsibility? *BJOG: An International Journal of Obstetrics & Gynaecology* 1985;92(1):1-3. doi: doi:10.1111/j.1471-0528.1985.tb01039.x
23. Mander R, Fleming V. Failure to progress: the contraction of the midwifery profession: Psychology Press 2002.
24. Kirkham M. The culture of midwifery in the National Health Service in England. *Journal of Advanced Nursing* 1999;30(3):732-39.
25. Prosser SJ, Barnett AG, Miller YD. Factors promoting or inhibiting normal birth. *BMC Pregnancy and Childbirth* 2018;18(1):241. doi: 10.1186/s12884-018-1871-5
26. Best A, Greenhalgh T, Lewis S, et al. Large-system transformation in health care: a realist review. *The Milbank Quarterly* 2012;90(3):421-56.
27. Pfadenhauer LM, Gerhardus A, Mozygemba K, et al. Making sense of complexity in context and implementation: the Context and Implementation of Complex Interventions (CICI) framework. *Implementation science* 2017;12(1):21.
28. McCourt C, Rayment J, Rance S, et al. Place of Birth and Concepts of Wellbeing: An Analysis from Two Ethnographic Studies of Midwifery Units in England. *Anthropology in Action* 2016;23(3):17-29.
29. Monitor. Moving healthcare closer to home: Literature review of clinical impacts. London: Monitor, 2015.
30. Hinton L, Dumelow C, Rowe R, et al. Birthplace choices: what are the information needs of women when choosing where to give birth in England? A qualitative study using online and face to face focus groups. *BMC Pregnancy and Childbirth* 2018;18(1):12. doi: 10.1186/s12884-017-1601-4
31. England N. National Maternity Review. Better Births; Improving Outcomes of Maternity Services in England. *NHS England, London* 2016
32. England N. Implementing Better Births: A resource pack for Local Maternity Systems. Publications Gateway Ref No. 06648: NHS England, 2017.

Corresponding Author

Dr Denis Walsh

Author Statement

Denis Walsh Chief Investigator, Associate Professor in Midwifery

Denis.walsh@ntlworld.com

- Principal author, drafting and revising content critically for important intellectual content, substantial contribution to the interpretation of data, final approval of the version to be published, agreement to be accountable for all aspects of the work in ensuring that questions related to the accuracy or integrity of any part of the work are appropriately investigated and resolved

Helen Spiby Co-Investigator, Professor in Midwifery

Helen.spiby@nottingham.ac.uk

For peer review only

- Drafting and revising content critically for important intellectual content, substantial contribution to the interpretation of data, final approval of the version to be published, agreement to be accountable for all aspects of the work in ensuring that questions related to the accuracy or integrity of any part of the work are appropriately investigated and resolved

Christine McCourt Co-Investigator, Professor in Maternal Health

Christine.mccourt.1@city.ac.uk

- Drafting and revising content critically for important intellectual content, substantial contribution to the interpretation of data, final approval of the version to be published, agreement to be accountable for all aspects of the work in ensuring that questions related to the accuracy or integrity of any part of the work are appropriately investigated and resolved

Celia Grigg Research Fellow

celia.grigg@xtra.co.nz

- Drafting and revising content critically for important intellectual content, substantial contribution to the interpretation of data, final approval of the version to be published, agreement to be accountable for all aspects of the work in ensuring that questions related to the accuracy or integrity of any part of the work are appropriately investigated and resolved

Dawn Coleby Senior Research Fellow

ac7301@coventry.ac.uk

- Drafting and revising content critically for important intellectual content, substantial contribution to the interpretation of data, final approval of the version to be published, agreement to be accountable for all aspects of the work in ensuring that questions related to the accuracy or integrity of any part of the work are appropriately investigated and resolved

Simon Bishop Co-Investigator, Senior Lecturer in Business School

Simon.Bishop@nottingham.ac.uk

- Drafting and revising content critically for important intellectual content, substantial contribution to the interpretation of data, final approval of the version to be published, agreement to be accountable for all aspects of the work in ensuring that questions related to the accuracy or integrity of any part of the work are appropriately investigated and resolved

For peer review only

Miranda Scanlon Co-Investigator, Service User

Miranda.Scanlon@city.ac.uk

- Drafting and revising content critically for important intellectual content, substantial contribution to the interpretation of data, final approval of the version to be published, agreement to be accountable for all aspects of the work in ensuring that questions related to the accuracy or integrity of any part of the work are appropriately investigated and resolved

Lorraine Culley Co-Investigator, Emeritus Professor of Social Science & Health

lac@dmu.ac.uk

- Drafting and revising content critically for important intellectual content, substantial contribution to the interpretation of data, final approval of the version to be published, agreement to be accountable for all aspects of the work in ensuring that questions related to the accuracy or integrity of any part of the work are appropriately investigated and resolved

Jane Wilkinson GP Commissioner

jane.wilkinson14@nhs.net

- Drafting and revising content critically for important intellectual content, final approval of the version to be published, substantial contribution to the interpretation of data, agreement to be accountable for all aspects of the work in ensuring that questions related to the accuracy or integrity of any part of the work are appropriately investigated and resolved

Lynne Pacanowski Head of Midwifery

homeofthepacs@gmail.com

- Drafting and revising content critically for important intellectual content, final approval of the version to be published, substantial contribution to the interpretation of data, agreement to be accountable for all aspects of the work in ensuring that questions related to the accuracy or integrity of any part of the work are appropriately investigated and resolved

Jim Thornton Professor of Obstetrics

Jim.Thornton@nottingham.ac.uk

For peer review only

- Drafting and revising content critically for important intellectual content, substantial contribution to the interpretation of data, final approval of the version to be published, agreement to be accountable for all aspects of the work in ensuring that questions related to the accuracy or integrity of any part of the work are appropriately investigated and resolved

Acknowledgements

We are extremely grateful to all who contributed to the study, including Heads of Midwifery for their support to the Stage 1 mapping phase; all who contributed during through interviews or focus groups to the Stage 2 case study phase and participants at the Stage 3 stakeholder Event.

We would also like to thank the members of the Service User Reference Panel for their invaluable contributions: Melissa Thomas, Leanne Stamp, Samantha Foulke, Joanne Whyler

We would like to thank Dr Juliet Rayment for her contribution to the media analysis of FMUs that had closed and ‘Which? Birth Choice’ for allowing us access to their data.

We would also like to acknowledge and thank Sheila Adamson at the University of Nottingham for all her work as the Research Secretary over 24 months.

Data Sharing Statement*:* Data are available in a public, open access repository:

https://www.journalslibrary.nihr.ac.uk/programmes/hsdr/140428/#/

For peer review only

Table 1

No.	Topic	Item
	**Title and abstract**	
S1	Title	Concise description of the nature and topic of the study identifying the study as qualitative or indicating the approach (e.g., ethnography, grounded theory) or data collection methods (e.g., interview, focus group) is recommended
S2	Abstract	Summary of key elements of the study using the abstract format of the intended publication; typically includes background, purpose, methods, results, and conclusions
	**Introduction**	
S3	Problem formulation	Description and significance of the problem/phenomenon studied; review of relevant theory and empirical work; problem statement
S4	Purpose or research question	Purpose of the study and specific objectives or questions
	**Methods**	
S5	Qualitative approach and research paradigm	Qualitative approach (e.g., ethnography, grounded theory, case study, phenomenology, narrative research) and guiding theory if appropriate; identifying the research paradigm (e.g., postpositivist, constructivist/ interpretivist) is also recommended; rationale[b]
S6	Researcher characteristics and reflexivity	Researchers' characteristics that may influence the research, including personal attributes, qualifications/experience, relationship with participants, assumptions, and/or presuppositions; potential or actual interaction between researchers' characteristics and the research questions, approach, methods, results, and/or transferability
S7	Context	Setting/site and salient contextual factors; rationale[b]
S8	Sampling strategy	How and why research participants, documents, or events were selected; criteria for deciding when no further sampling was necessary (e.g., sampling saturation); rationale[b]
S9	Ethical issues pertaining to human subjects	Documentation of approval by an appropriate ethics review board and participant consent, or explanation for lack thereof; other confidentiality and data security issues
S10	Data collection methods	Types of data collected; details of data collection procedures including (as appropriate) start and stop dates of data collection and analysis, iterative process, triangulation of sources/methods, and modification of procedures in response to evolving study findings; rationale[b]
S11	Data collection instruments and technologies	Description of instruments (e.g., interview guides, questionnaires) and devices (e.g., audio recorders) used for data collection; if/how the instrument(s) changed over the course of the study
S12	Units of study	Number and relevant characteristics of participants, documents, or events included in the study; level of participation (could be reported in results)
S13	Data processing	Methods for processing data prior to and during analysis, including transcription, data entry, data management and security, verification of data integrity, data coding, and anonymization/deidentification of excerpts
S14	Data analysis	Process by which inferences, themes, etc., were identified and developed, including the researchers involved in data analysis; usually references a specific paradigm or approach; rationale [b]
S15	Techniques to enhance trustworthiness	Techniques to enhance trustworthiness and credibility of data analysis (e.g., member checking, audit trail, triangulation); rationale[b]
	**Results/findings**	
S16	Synthesis and interpretation	Main findings (e.g., interpretations, inferences, and themes); might include development of a theory or model, or integration with prior research or theory
S17	Links to empirical data	Evidence (e.g., quotes, field notes, text excerpts, photographs) to substantiate analytic findings
	**Discussion**	
S18	Integration with prior work, implications, transferability, and contribution(s) to the field	Short summary of main findings; explanation of how findings and conclusions connect to, support, elaborate on, or challenge conclusions of earlier scholarship; discussion of scope of application/ generalizability; identification of unique contribution(s) to scholarship in a discipline or field
S19	Limitations	Trustworthiness and limitations of findings
	**Other**	
S20	Conflicts of interest	Potential sources of influence or perceived influence on study conduct and conclusions; how these were managed
S21	Funding	Sources of funding and other support; role of funders in data collection, interpretation, and reporting

[a]The authors created the SRQR by searching the literature to identify guidelines, reporting standards, and critical appraisal criteria for qualitative research; reviewing the reference lists of retrieved sources; and contacting experts to gain feedback. The SRQR aims to improve the transparency of all aspects of qualitative research by providing clear standards for reporting qualitative research.

[b]The rationale should briefly discuss the justification for choosing that theory, approach, method, or technique rather than other options available, the assumptions and limitations implicit in those choices, and how those choices influence study conclusions and transferability. As appropriate, the rationale for several items might be discussed together.

ACADEMIC MEDICINE

[Standards for Reporting Qualitative Research: A Synthesis of Recommendations](#)

O'Brien, Bridget C.; Harris, Ilene B.; Beckman, Thomas J.; Reed, Darcy A.; Cook, David A.

Academic Medicine89(9):1245-1251, September 2014.

doi: 10.1097/ACM.0000000000000388

Standards for Reporting Qualitative Research (SRQR)a

Wolters Kluwer

Copyright © 2019 by the Association of American Medical Colleges

Wolters Kluwer

Thank You

This PowerPoint document contains the images that you requested.

Copyright Notice

All materials on this Site are protected by United States copyright law and may not be reproduced, distributed, transmitted, displayed, or otherwise published without the prior written permission of Wolters Kluwer. You may not alter or remove any trademark, copyright or other notice.

However, provided that you maintain all copyright, trademark and other notices contained therein, you may download material (one machine readable copy and one print copy per page) for your personal, non-commercial use only. Please refer to this link for further information on how to
apply for permission for re-use

Any information posted to discussion forums (moderated and un-moderated) is for informational purposes only. We are not responsible for the information or the result of its practice.

BMJ Open

Factors influencing the utilisation of free-standing and alongside midwifery units in England: A Qualitative Research Study

Journal:	*BMJ Open*
Manuscript ID	bmjopen-2019-033895.R1
Article Type:	Original research
Date Submitted by the Author:	18-Dec-2019
Complete List of Authors:	Walsh, Denis; University of Nottingham, School of Health Sciences<br>Spiby, Helen; University of Nottingham Faculty of Medicine and Health Sciences<br>McCourt, Christine; City University<br>Grigg, Celia<br>Coleby, Dawn; De Montfort University<br>Bishop, Simon; University of Nottingham<br>Scanlon, Miranda; City University<br>Culley, Lorraine; De Montfort University<br>Wilkinson, Jane<br>Pacanowski, Lynne<br>Thornton, Jim; University of Nottingham
**Primary Subject Heading**:	Obstetrics and gynaecology
Secondary Subject Heading:	Obstetrics and gynaecology, Health services research
Keywords:	OBSTETRICS, Organisation of health services < HEALTH SERVICES ADMINISTRATION & MANAGEMENT, Health policy < HEALTH SERVICES ADMINISTRATION & MANAGEMENT

SCHOLARONE™
Manuscripts

I, the Submitting Author has the right to grant and does grant on behalf of all authors of the Work (as defined in the below author licence), an exclusive licence and/or a non-exclusive licence for contributions from authors who are: i) UK Crown employees; ii) where BMJ has agreed a CC-BY licence shall apply, and/or iii) in accordance with the terms applicable for US Federal Government officers or employees acting as part of their official duties; on a worldwide, perpetual, irrevocable, royalty-free basis to BMJ Publishing Group Ltd ("BMJ") its licensees and where the relevant Journal is co-owned by BMJ to the co-owners of the Journal, to publish the Work in this journal and any other BMJ products and to exploit all rights, as set out in our licence.

The Submitting Author accepts and understands that any supply made under these terms is made by BMJ to the Submitting Author unless you are acting as an employee on behalf of your employer or a postgraduate student of an affiliated institution which is paying any applicable article publishing charge ("APC") for Open Access articles. Where the Submitting Author wishes to make the Work available on an Open Access basis (and intends to pay the relevant APC), the terms of reuse of such Open Access shall be governed by a Creative Commons licence – details of these licences and which Creative Commons licence will apply to this Work are set out in our licence referred to above.

Other than as permitted in any relevant BMJ Author's Self Archiving Policies, I confirm this Work has not been accepted for publication elsewhere, is not being considered for publication elsewhere and does not duplicate material already published. I confirm all authors consent to publication of this Work and authorise the granting of this licence.

For peer review only

Title Page

Title:

Factors influencing the utilisation of free-standing and alongside midwifery units in England: A Qualitative Research Study

Authors:

Dr Denis Walsh, Retired Associate Professor (Lead and Corresponding Author)

University of Nottingham

Nottingham

UK

Postal Address:

116 Letchworth Rd, Leicester LE3 6FH

Telephone: 07905735777

Email: Denis.walsh@ntlworld.com

Helen Spiby, Co-Author, Professor in Midwifery

University of Nottingham

Nottingham

UK

Christine McCourt, Co-Author, Professor in Maternal Health

City, University of London

UK

Dr Celia Grigg, Co-Author, Research Fellow

University of Auckland

Auckland

New Zealand

Dr Dawn Coleby, Co-Author, Early Career Academic Fellow

De Montfort University

For peer review only

Leicester

UK

Dr Simon Bishop Co-Author, Senior Lecturer in Business School

University of Nottingham

Nottingham

UK

Dr Miranda Scanlon Co-Author, Senior Research Fellow and Service User

City, University of London

UK

Lorraine Culley Co-Author, Emeritus Professor of Social Science & Health

De Montfort University

Leicester

UK

Jane Wilkinson, Co-Author, GP Commissioner

Wirral Community NHS Foundation Trust

Liverpool

UK

Lynne Pacanowski, Co-Author, Retired Head of Midwifery

Guys & St Thomas' NHS Trust

London

UK

Jim Thornton, Co-Author, Professor of Obstetrics

University of Nottingham

Nottingham

UK

For peer review only

Key Words: Obstetrics, Qualitative Research, Midwifery Units

Word Count: 4688

For peer review only

ABSTRACT: Factors influencing the utilisation of free-standing and alongside midwifery units in England: A Qualitative Research Study

Objective: To identify factors influencing the provision, utilisation and sustainability of midwifery units (MUs) in England

Design: Case studies, using individual interviews and focus groups, in six NHS Trust maternity services in England

Setting & Participants NHS maternity services in different geographical areas of England

Maternity care staff and service users from 6 NHS Trusts: 2 Trusts where more than 20% of all women gave birth in MUs, 2 Trusts where less than 10% of all women gave birth in MUs and 2 Trusts without MUs. Obstetric, midwifery and neonatal clinical leaders, managers, service user representatives and commissioners were individually interviewed (n=57). Twenty-six focus groups were undertaken with midwives (n=60) and service users (n=52).

Main Outcome Measures: Factors influencing MU use

Findings: The study findings identify several barriers to the uptake of MUs. Within a context of a history of obstetric-led provision and lack of decision-maker awareness of the clinical and economic evidence, most Trust managers and clinicians do not regard their MU provision as being as important as their obstetric unit (OU) provision. Therefore, it does not get embedded as an equal and parallel component in the Trust's overall maternity package of care. The analysis illuminates how implementation of complex interventions in health services is influenced by a range of factors including the medicalisation of childbirth, perceived financial constraints, adequate leadership and institutional norms protecting the status quo.

Conclusions: There are significant obstacles to MUs reaching their full potential, especially free-standing midwifery units (FMUs). These include the lack of commitment by providers to embed MUs as an essential service provision alongside their OUs, an absence of leadership to drive through these changes and the capacity and willingness of providers to address women's information needs. If these remain unaddressed, childbearing women's access to MUs will continue to be restricted.

Strengths and limitations of this study

1. The richness and breadth of data captured across multiple case study sites with contrasting organisational characteristics

2. The focus groups generated discussion and insight unlikely to be obtained by individual interviews and were particularly effective in comparing service user perspectives with provider perspectives from within the same case.

3. We were unable to get access to Trust documentation regarding MU policies and organisation which may have helped triangulate data from the interviews and focus groups.

4. We were not able to include service users from all communities in the focus groups as we did not have translation services.

Funding

This work was supported by the National Institute for Health Research (NIHR) Health Services and Delivery Research programme (Ref: 14/04/28).

Competing Interest Statement

Professor Thornton reports being a member of the HTA and EME Boards. Dr Scanlon reports grants from NIHR, during the conduct of the study; personal fees from "WHICH?", grants from NIHR, personal fees from National Perinatal Epidemiology Unit, personal fees from Rod Gibson Associates Ltd, personal fees from Midwifery Unit Network, outside the submitted work. No other competing interests have been declared.

For peer review only

INTRODUCTION

Since 1993, maternity care policy in England has promoted women's choice of place of birth. This became the national choice guarantee in Maternity Matters in 2007[1] which stipulated women should have three options: birth in a hospital (obstetric unit or OU), birth in two types of midwifery unit (MU) - either alongside (AMU) or freestanding (FMU) - or birth at home. MUs are birthing facilities for 'low risk' women run by midwives, though in the English context unlike other parts of the world, very few provide continuity of carer through all phases of maternity care. AMUs are attached to existing hospitals with obstetric units while FMUs are geographically separate. The Birthplace in England cohort study[2] reported that outcomes for low risk pregnant women were better and costs reduced if birth occurred in MUs, both AMUs and FMUs, rather than OUs. For example, having a baby in a MU reduced caesarean section rates by two thirds, while there was no difference in adverse neonatal outcomes. These findings have since been supported by a systematic review of international evidence, which drew similar conclusions[3].

The most recent National Institute for Health and Care Excellence (NICE IP 390) guideline on intrapartum care therefore recommend MUs for low risk women, i.e. women without significant health risk factors who are predicted to have a normal labour and birth[4]. Sandall and colleagues' research suggests this could be around 45% of all birthing women by the onset of labour[5]. Therefore 36% of this group could be expected to give birth in MUs, allowing for a 20% intrapartum transfer rate found in the Birthplace in England study[2]. However, despite the advantages of MUs, the National Audit Office (NAO) found in 2013 that only 11% of women gave birth in those settings while the vast majority continue to give birth in OUs[6]. In addition, MUs were not equally distributed across the country[6]. A third of NHS Trusts had no MUs, and those that did, were frequently underutilised with less than 10% of all births occurring in them. If 20% of births occurred in MUs, savings to the NHS maternity budget could be around £85 million/year, projecting from average cost differences[7]. This represents a 3% saving on the current annual budget of £2.6 billion for maternity care[8].

The NICE intrapartum guidelines and maternity care policy emphasis on patient or consumer choice are in line with the direction of national policy across wider areas of healthcare. Midwifery units could be considered an example of a complex health service 'intervention' that is a change in organisation models, based on best clinical evidence, that require a systemic, multi-stakeholder approach to implementation. A range of prior studies have highlighted challenges in the implementation of health policies and evidence of this nature[9-11].

There has been no specific research investigating the reasons for the highly varied rates of MU provision across England since publication of the NAO survey. Our research was conducted to explore the reasons for these anomalies in the provision of MUs in England. The principal objectives of the study were to describe the configuration, organisation and operation of MUs in England and identify key barriers to the uptake of MU care. A three-phase mixed methods study incorporating a Mapping Survey (Phase 1), Comparative Case Studies (Phase 2) and a Stakeholder Workshop (Phase 3) was undertaken to answer these objectives. The national mapping of MUs and OUs nationally (including numbers and organisation) has already been reported[12].

The most significant finding of the mapping phase (which included all 134 NHS Trusts providing maternity services) was that, although the percentage of births in MUs has increased from 5% to 14% since the Birthplace in England study, that growth has occurred in AMUs[12]. This falls well short of the potential percentage of births in MUs of 36%, previously mentioned. The mapping phase also identified organisational processes within maternity services regarding MU access and utilisation. Two key findings were, firstly that 97% percent of AMU midwives and 50% of FMU midwives were moved regularly during shifts, usually to the OU. Staff shortage or 'capacity issues' on the OU were the primary reason given for MU closures, which occurred for 28% of AMUs and 39% of FMUs. Thus, some MU midwives were providing care for low risk women in OUs while AMUs and FMUs stood vacant. AMUs that were underutilised (i.e. <10% of births) were closed three times as frequently as AMUs where >20% of women gave birth. Secondly, AMU admission rates were facilitated in some settings by maternity services operating an opt-out policy i.e. women who met eligibility criteria were defaulted to them unless they opted otherwise, rather than a more traditional OU opt-out policy. Of the high-performing Trusts with AMUs, 73% had an opt-out policy compared with only 14% of the low-performing Trusts with AMUs.

Here we report the methods and findings for phase 2 of our overall study. The objective was to identify factors influencing the provision, utilisation and sustainability of midwifery units, and to understand in more depth the picture obtained in the mapping survey[12].

METHODS

We conducted qualitative case studies to understand and compare maternity services with different levels of progress in the implementation of MUs. Based on our mapping survey findings of 97 AMUs and 61 FMUs in England, we chose six case-study sites to study in-depth. Two were high-performing (our definition: MUs achieving 20% or more of all local facility-based births), two were low-performing (MUs achieving 10% or less of all local births) and two sites had no MUs. From 82 of the 134 Trusts meeting these eligibility criteria, in addition, we chose a mix of metropolitan and rural areas from different geographical areas with varying sizes of service and configurations. Data collection from each site involved: individual interviews with senior managerial and clinical midwives, obstetricians and neonatologists, Trust CEOs, commissioners and service user representatives in each case study site (n=57); 13 focus groups with clinical midwives (n=60); 13 focus groups with women who had recently used maternity services (n=52). Local heads of midwifery assisted the researcher in the identification of Trust clinical and managerial leadership, who were approached by the researchers directly. The midwifery leaders also facilitated the distribution of the invitation to participate in focus groups to their midwifery workforce. The service user representatives assisted researchers with identifying potential groups and venues to advertise the service user focus groups. Additionally, the research team independently approached community centres to advertise the groups. All participants provided written consent. Interviews and midwives focus groups were conducted by research staff, and service user focus groups were co-facilitated by research staff and a member of the project's service user reference group. Interview guides were developed and piloted for all individual interviews and focus groups. Focus group size ranged from 3 to 7 people. All focus groups and interviews were recorded and professionally transcribed.

For peer review only

The women's focus groups were analysed by open coding, followed by thematic distillation as outlined by Braun and Clarke[13]. All remaining focus groups and interviews were analysed with the Consolidated Framework for Implementation Research (CFIR), which provides a list of domains previously found to impact on the process of implementing evidence at an organisational level across healthcare organisations[14] CFIR utilises five domains[15]: 1) the 'outer' wider health system (policies) and society (norms), 2) the characteristics of the individuals involved (beliefs, preferences), 3) the 'inner' context of the relevant organisations i.e. NHS Trusts – their culture, networks etc, 4) the context and nature of the 'intervention' – in this case MUs and 5) the process of change (implementing the intervention). Each of these domains has a number of constructs which findings were mapped to. Though this process is largely abductive i.e. moving iteratively between inductive and deductive modes, the CFIR accommodates the creation of additional constructs inductively from the data. On completion of this, analysis proceeded by comparing and contrasting themes from the women's focus groups with the CFIR constructs 'within cases' and then on a 'cross-case' basis. Cross case analysis was guided by the over-arching question of why some services were successful in opening, utilising and sustaining MUs and others were not.

Following analysis, we convened a meeting attended by 56 stakeholders from across England comprising provider, commissioner, education and service user constituencies, for phase three. Findings were presented, and discussion groups identified a set of priority actions to help services to increase the provision and uptake of MUs. The detail of this phase is not reported here.

Ethical approval was granted for phase 2 of the study the West Midlands - Solihull Research Ethics Committee (IRAS ID 200356) as phase 1 and 3 were deemed service development.

Public and Patient Involvement

Public involvement was integrated into the study throughout all phases including project design, implementation, management and dissemination. One of the Co-Investigators was a service user and contributed to the original idea for the research and to developing the research protocol. Four service users were recruited to a service user reference group from an established local service user maternity network. This group reviewed all aspects of the study design, including the study documents. Group members advised on approaches to achieve recruitment of women into focus groups, and co-facilitated the women's focus groups, with a member of the research staff, at the six case study sites. They also co-presented the preliminary findings at the Stakeholder Workshop and co-facilitated group discussions at this event. They will also be involved in the dissemination of findings via their Facebook groups.

Additional aspects of the methods, more detail on the analytical approach across all three phases, reflections on the utility of the CFIR, sample sizes and composition will be available electronically in the Final NIHR Report to be published on the 31st of January 2020[16].

FINDINGS

The case study analysis distilled key themes that need addressing if English maternity services are to maximise the provision, utilisation and sustainability of MUs and therefore accrue their clinical benefits. This synthesis of the analysis will be reported under the various domains of the CFIR. Table 1 is illustrative of the process.

Table 1: Themes Mapped to CFIR Domains

Key cross-cutting themes mapped on to CFIR framework							
**CFIR Domains & linked Constructs**		**Cross cutting themes**					
**I. INTERVENTION CHARACTERISTICS**		**Culture and beliefs about the intervention**	**Resources and Priorities**	**Organisation**	**Staffing**	**Leadership**	**Change**
A	Intervention Source						
B	Evidence Strength & Quality						
C	Relative Advantage						
D	Adaptability						
E	Trialability						
F	Complexity						
G	Design Quality & Packaging						
H	Cost						
**II. OUTER SETTING**							
A	Patient Needs & Resources						
B	Cosmopolitanism						
C	Peer Pressure						
D	External Policy & Incentives						
**III. INNER SETTING**							
A	Structural Characteristics						
B	Networks & Communications						

For peer review only

C	Culture						
D	Implementation Climate						
1	Tension for Change						
2	Compatibility						
3	Relative Priority						
4	Organizational Incentives & Rewards						
5	Goals and Feedback						
6	Learning Climate						
E	Readiness for Implementation						
1	Leadership Engagement						
2	Available Resources						
3	Access to Knowledge & Information						
**IV. CHARACTERISTICS OF INDIVIDUALS**							
A	Knowledge & Beliefs about the Intervention						
B	Self-efficacy						
C	Individual Stage of Change						
D	Individual Identification with Organization						
E	Other Personal Attributes						
**V. PROCESS**							
A	Planning						
B	Engaging						

1	Opinion Leaders						
3	Champions						
4	External Change Agents						
C	Executing						
D	Reflecting & Evaluating						

Outer Setting

We found strong institutional and societal pressure (risk and litigation policies, fiscal constraints) to maintain OUs as the core focus of maternity care, positioning MUs as a lesser priority and an optional extra. This involved a number of elements, including legal and governance frameworks, professional hierarchies and resource flows, which contributed to the dominance of OU care. Particularly important were perceptions of appropriate approaches to managing risk, present in the responses of representatives from all professional groups, which had not been adjusted in the light of the clinical evidence.

“There's also the potential clinical risks of people giving birth in those areas (AMUs). And we had an unfortunate death about three years ago..” [Obstetrician]

“There might be a degree of fear that if people started saying that, you can go in there (to the MU), you are constantly reminded that women have to be told the risks. ...because of the litigation now.” [Midwife in focus group]

No professionals raised concerns about the increased risk of medical interventions associated with women giving birth in OUs.

Factors in the ‘outer setting’ of midwifery could be seen as contributing to a ‘medical’ view of childbirth that shaped perceptions of where birth should be situated. This was highlighted in women’s focus groups:

“..we’ve been become accustomed to birth taking place in hospital (OUs) and to step outside that model you’ve got to face your family and peers and actually have a good reason why you want to birth outside that accepted model…hospital is perceived as safest, the ‘just in case’ option..”

Another factor to emerge from interviews, especially from service providers, was budget constraints. Financial cutbacks within Trusts were mentioned across all sites as frustrating the development of MUs:

“I think the whole financial situation within the Trust at the moment is a driver. ... Unfortunately, all our finance team will only see is the figure at the bottom of the page. ...it is a sort of finance driven organisation and you’re forever trying to find ways of saving money, cutting costs, etc” [Midwifery Manager]

For peer review only

All respondents appeared to accept the need for Trusts to save money as a ‘fait accompli’ and the unaffordability of MUs as a ‘fact’ as typified by the phrase ‘*we’re in a period of austerity now*’ and positioned maternity as competing and losing out to other services. The findings indicated little awareness of the evidence on cost-effectiveness of MU facilities.

Characteristics of Individuals

Closely related to a medicalised view of childbirth, we found mixed beliefs among individuals about the efficacy of MUs, with pockets of strong scepticism across high and low uptake sites. In many instances, these attitudes took precedence over opposing views emanating from the clinical evidence. Antipathy towards MUs was particularly strong in the case of FMUs, in relation to which several common assumptions were noted. These included the perceived superior safety of the medical model, that FMUs and AMUs offer essentially an identical service and that FMUs are not popular with women:

I think majority of women and all my friends will opt for an alongside MU, because most women do want the option of midwifery led but if anything goes wrong they just want to go down that corridor, through that door. [Midwifery Manager]

Many midwives, especially in sites with no MUs, were reported as actively resisting the development of an FMU:

“..they (the midwives) were completely horrified at the idea of having a standalone midwifery-led unit” [Midwifery Manager]

While variations of this attitude could be found across all sites, within high performing sites we did find existing AMU and FMU ‘champions’ who saw themselves as contributing to the ‘mission’ or ‘vision’ of midwifery led birth:

“*The vision is to up the numbers, so we have the quality boards, and we are aiming to increase the deliveries in the midwifery led unit, and home deliveries. …we are continuously striving to increase it.”* [Midwife]

Inner Setting

We found that collaboration between MUs and OUs was important for the successful embedding of the MU model, and pockets of excellent collaborative relations were reported within high performing sites. More commonly, this did not occur, creating an ‘us and them’ atmosphere as illustrated by this segment of a focus group transcript between an FMU midwife and the facilitator:

Int: I went on a transfer, and the reception I got was non-existent.
Fac: What do you mean?
Int: There was nobody waiting…there wasn’t a cot in the room, no midwife came, I had to find somebody.
Fac: But they’re always told ahead that you’re coming?
Int: Oh yeah, they know you’re coming. I’ve been greeted with ‘oh, here comes another failure from FMU’.

For peer review only

We also found evidence in some Trusts of a culture of marginalising and undervaluing of FMUs. As a result, several FMUs were under threat of closure, even in high-performing sites. The two dominant rationales for closure were that they are under-used and too expensive as illustrated by these quotes:

"Well it (FMU) is small and we do have to understand how viable it (FMU) is because you can't spread yourself so thin. So it (FMU) is difficult to manage because we're covering so many other areas, and the birth rates numbers are very low" [Manager]

"If you spoke to any of the consultants I am sure they would say it [FMU] should be closed because it's a waste of money. And it's an unfair allocation of resources, in a relatively resource poor environment." [Manager]

In addition, we found evidence that FMUs can be subjected to a mixture of managerial neglect and authoritarian control from their host Trusts. An FMU manager said:

"They (Trust management) always pay us lip service… don't promote us'…we've been fighting for a year to get a video on the Trust website, of a tour of our birth centre…. You do feel like the poor relation". [Focus Group Midwife]

This manifested in several contradictory messages coming from some Trusts. We found examples of all of the following: using FMUs to solve capacity crises at times of peak activity while threatening them with closure at other times; restricting opportunities for FMU staff to promote their services as illustrated in the quote above; FMU staff not being consulted on strategic changes that impacted on them as this excerpt from an individual interview revealed:

"To hear the news about the closure (of the FMU) on the TV rather than from the organisation was terrible, so it makes them, you know, lose confidence. [FMU Manager].

Intervention Characteristics

Embedding MU provision was perceived as presenting a number of challenges. MUs are intended to provide care to low risk women, where midwives can practice the skills of normal midwifery. However, a number of midwife respondents felt that practicing within them required different skills and a level of confidence, which they were not well prepared for.

"Because everyone has worked in such a high-risk environment, you become deskilled to an extent, and feel a bit apprehensive about normal birth… you know, trusting that women can have babies low risk." [Focus Group Midwife]

Midwifery managers and midwives in our study recommended mandatory training in normal birth skills to address this concern. Linked to a perceived deficit in skills and arguably more influential was a lack of confidence amongst midwives to make decisions in MU settings where midwives are more autonomous, as illustrated from this midwife focus group:

For peer review only

"One of the effects that that has had, is that it has decreased a lot of the midwives' confidence in this unit, of providing low risk care, because they don't have the environment, a consistent one, in which to do it properly…. when you're on labour ward you become over reliant on the doctors."

Process

There were considerable differences across sites in the process of implementing and developing MUs. Leadership emerged as key to successful implementation.

"it's crucial to have an inspirational leader. If you don't have somebody at the very top who is passionate about it (MUs) happening, it won't happen. And they must cascade, get everybody onboard." [Midwives Focus Group]

"a charismatic leader to kind of bring it together…unless you've got that then I think it's quite hard to bring it to fruition." [Manager]

Continuity of leadership contributing to organisational memory was also stressed:

"I think the birth centres are being used less at the moment, and that does seem to coincide with a change of leadership." [Midwives Focus Group]

Only a few sites had an active policy of the ongoing promotion of MUs to their local women to increase and sustain their utilisation:

"So you have to do a lot of positive promotion, you have to get out there. And you're almost selling a product. And that's how we saw it. So we did lots of promotional events, and got lots in the press, about the opening of the FMU." [Manager]

Successful implementation was also dependent on a clear clinical pathway from the beginning of pregnancy until the onset of labour. For example, there was a wide variation in the information women had, or were given, about MUs - within and between Trusts. The majority of women in the focus groups reported not receiving information. Participants from five of the six case study Trusts mentioned not being given information about the local MUs (including the two which have more than 20% of women giving birth in a MU).

"Well it's just that nobody gave us the information about it [MUs]. That's the main thing. I didn't know nothing about it. I didn't even know it even existed." [Women's Focus Group]

Women expressed concerns about the place of birth booking process, such as whether it was necessary to decide at the beginning of pregnancy, how it was done, and if it was possible to change your mind.

"I wasn't aware that you had to decide before you went in for your booking appointment, so I was asked on the spot and I didn't know. But the midwife said that you have to choose now because they have to book the hospital in advance." [Women's Focus Group]

DISCUSSION

For peer review only

This research has illuminated why MUs are underused in England and still not available in many NHS Trusts. The central challenge in all case study sites was introducing and sustaining what was still perceived as an alternative configuration (MUs) into an existing and embedded mainstream, 'taken for granted' model (OUs) which has been in place for the past five decades. OUs are the default place of birth for the vast majority of women in England, regardless of women's risk profiles. Utilising the domains of the CFIR, our findings show how several multiple external (outer context), and internal (inner context) factors, alongside personal beliefs of key players, intrinsic features of MU services and the process of change itself combine to reinforce the status quo and slow the growth of MUs.

Coxon[17 18] and Scamell[19] argue that the construction of birth as risky in policy initiatives and by service providers over recent decades has shaped women's preference for birth in OUs. Birth as a risky endeavour is a by-product of the medicalisation of childbirth over a similar period that has seen caesarean section rates rise exponentially[20 21]. As Coxon demonstrated, if women's first experience of birth is in a hospital labour ward, they are likely to choose the same for subsequent births[17]. What this study has illustrated is that professional perceptions of what is risky and how risk should be managed can be out of step with evidence – in this case, the evidence on the safety of different birth settings[2 3]

Despite national guidelines based on extensive evidence recommending MUs for women at low obstetric risk, we found that managers, midwives and clinicians in provider settings harboured considerable ambivalence about the safety of MUs. Research has shown that personal belief can moderate evidence[22] and is a key variable to address in systematic reviews of what facilitates the translation of evidence into practice[23 24]. FMUs were especially vulnerable to negative beliefs about their efficacy even though they pre-date AMUs by decades, albeit under the title of maternity homes or GP units. Though AMUs are a relatively new phenomenon, there has been an exponential increase in their use over the past 6 years, even if still at a low overall level, which could reflect the broader bias favouring the embedded system of OUs as AMUs are co-located with them.

Financial constraints within Trusts were often seen as limiting the development of MUs. While economic evaluations suggest the overall economic outcomes of increasing births in MUs is positive[25], the start-up costs were seen as a barrier, and the longer-term savings from lower morbidity in the target population that accrue across the health system were not recognised. In a climate of scarcity, new ways of structuring care must demonstrably save money, or at least, be perceived to, in the short term.

A defining characteristic of MUs as an intervention is that their functioning is entirely dependent on midwives because they are midwife-led and managed. Skills in managing normal labour and birth and decision-making autonomy are prerequisites for practise in this setting. Our findings highlighted a lack of midwifery confidence and skill that can be traced back to the training and practice of UK midwives within predominantly obstetric-led services. Numerous surveys and papers have demonstrated this over the last 30 years since Robinson's pioneering research on the loss of traditional midwifery skills in the 1980s[26-29].

Our findings pinpoint the importance of leadership to the process of managing organisational change of this magnitude. Best et al's realist review of large system transformation of health services[30] found that blending designated leadership (someone in charge of the programme) with distributed leadership (professionals/partner organizations sharing responsibility for delivering it) was the most successful at embedding and sustaining change. For the successful

For peer review only

development and operationalisation of MUs, leadership needs to be exercised vertically via the layers of organisational hierarchy and horizontally across different professional groups; and at each of these levels, designated leadership and distributive leadership should be combined. An important component of leadership was the identification and subsequent impact of having 'champions'. Champions of MUs were either clinicians, managers or service users who were highly influential in promoting the service and recruiting support for it. Designated leaders working with champions were better at establishing clinical pathways for women to optimise access and utilisation of MUs. This included user friendly information to promote the choice of MUs, adopting an opt-out mechanism for AMUs and employing a consultant midwife to oversee and develop MUs.

A final issue illuminated by this study was the finding that despite arguments posited by service managers in relation to lack of demand, the majority of women in our focus groups reported lack of awareness of these services and lack of information provision about their options. This echoes the findings of Rayment et al in relation to women's access to MUs in England[31] and Henshall et al's systematic review, which highlighted professionals lack of skills and confidence in providing information, in a context where such services continue to be viewed as alternative[32].

Our findings help explain the difficulty moving away from the existing status-quo. Under each of the domains of the CFIR, the study identified issues that would appear to slow the growth of MUs. The current constitution of healthcare organisations, the policy environment, aspects of training, as well as complexities in the nature and process of change together work to maintain the dominance of OUs for birth. The study findings address the specific challenges for maternity care but also illuminate wider issues relevant to implementation science in health.

The strength of this comparative case study methodology is the richness and breadth of data captured across multiple sites with differing organisational characteristics. In addition, focus groups generated discussion and insight unlikely to be obtained by individual interviews. They were particularly effective in comparing service user perspectives with provider perspectives from within the same case. Inevitably, we were not able to include a full range of service users in the focus groups as we did not have translation services. Despite this, we did have BAME (Black, Asian and Minority Ethic) representation in some of the focus groups.

IMPLICATIONS

The key implication of this research is that, in many areas of England, women at low risk of complications do not have access to the maternity care that evidence shows is most suitable for them, because of the factors highlighted in this paper.

The importance of leadership was a principal finding from our case studies as it is a critical factor in the normalisation of an intervention to the point where it is no longer appraised as marginal but becomes incorporated and understood as a core part of the service[33].

It was clear from our study that inequality of access to information is primarily a matter of women not being given information about the option of MU care. Having an opt-out policy for FMUs should also be explored. FMUs have the additional advantage of being a more local provision for some women and therefore meeting the wider health service principle of

For peer review only

moving care closer to home[34]. In addition women may benefit from a higher quality of information about place of birth options and evidence, provided at different stages of the pregnancy[35].

The increase in the overall number of MUs since 2010 is due to the opening of AMUs. Trusts also need to value their FMU(s) as central to the broader maternity service provision and an important choice for low risk women. In particular, the common perception that FMUs are a financial burden unless operating at maximum capacity needs to be challenged as the available evidence suggests they are cheaper than supporting the same women to birth in an OU, even when the MU is operating at around 30% capacity. This is because health economists factored in the savings they generate in reduced intervention and maternal morbidity[7 25]. FMU facilities could also be used more extensively for other outpatient services and could arguably operate as part of a community hub as envisioned by the Implementing Better Births policy document[36 37].

CONCLUSIONS

NICE Intrapartum Care Guidance (IP390) first recommended birth in MUs for low risk women in 2014, but their potential for women across England is not being realised. This is because of the challenge of embedding them into the existing hospital-based OU model, that has been in place for several decades. Changing the status quo requires leadership from both commissioners and providers and a clearly articulated belief in the value of MUs, exercised through committing resources, streamlining care pathways and ongoing promotion to service users.

REFERENCES

1. Britain G. Maternity matters: choice, access and continuity of care in a safe service: Department of Health 2007.
2. Brocklehurst P, Hardy P, Hollowell J, et al. Perinatal and maternal outcomes by planned place of birth for healthy women with low risk pregnancies: the Birthplace in England national prospective cohort study. *British Medical Journal* 2011;343(7840):d7400.
3. Scarf VL, Rossiter C, Vedam S, et al. Maternal and perinatal outcomes by planned place of birth among women with low-risk pregnancies in high-income countries: A systematic review and meta-analysis. *Midwifery* 2018;62:240-55. doi: https://doi.org/10.1016/j.midw.2018.03.024
4. NICE NIoHE. Intrapartum Care: Care of healthy women and their babies during childbirth. Clinical Guideline 190 Methods, evidence and recommendations. 2 ed. London: NICE, 2014.
5. Sandall J, Murrells T, Dodwell M, et al. The efficient use of the maternity workforce and the implications for safety and quality in maternity care: a population-based, cross-sectional study. *Health Serv Deliv Res* 2014;2(38) doi: 10.3310/hsdr02380
6. NAO. National Audit Office (2013) Maternity services in England. London: NAO. London, 2013.
7. Schroeder E, Petrou S, Patel N, et al. Cost effectiveness of alternative planned places of birth in woman at low risk of complications: evidence from the Birthplace in England national prospective cohort study. *BMJ: British Medical Journal* 2012;344
8. Walsh D, Spiby H, McCourt C, Bishop S, Dodwell M, Culley L, Pacanowski L, Wilkinson J, Thornton J. HS & DR: NIHR, 2014:122.
9. Greenhalgh T, Papoutsi C. Spreading and scaling up innovation and improvement. *BMJ* 2019;365:l2068.
10. May C, Finch T, Mair F, et al. Understanding the implementation of complex interventions in health care: the normalization process model. *BMC Health Services Research* 2007;7(1):148.

For peer review only

11. Hasson H. Systematic evaluation of implementation fidelity of complex interventions in health and social care. *Implementation Science* 2010;5(1):67.
12. Walsh D, Spiby H, Grigg CP, et al. Mapping midwifery and obstetric units in England. *Midwifery* 2018;56(Supplement C):9-16. doi: https://doi.org/10.1016/j.midw.2017.09.009
13. Braun V, Clarke V. Using thematic analysis in psychology. *Qualitative research in psychology* 2006;3(2):77-101.
14. Damschroder LJ, Hagedorn HJ. A guiding framework and approach for implementation research in substance use disorders treatment. *Psychology of Addictive Behaviors* 2011;25(2):194-205. doi: 10.1037/a0022284
15. Breimaier HE, Heckemann B, Halfens RJ, et al. The Consolidated Framework for Implementation Research (CFIR): a useful theoretical framework for guiding and evaluating a guideline implementation process in a hospital-based nursing practice. *BMC nursing* 2015;14(1):43.
16. D Walsh HS, C McCourt, D Coleby, C Grigg, S Bishop, M Scanlon, L Culley, J Wilkinson, L Pacanowski, J Thornton. Factors influencing utilisation of 'free-standing' and 'alongside' midwifery units for low-risk births in England: a mixed-methods study. *Health Serv Deliv Res* 2020;in press
17. Coxon K, Chisholm A, Malouf R, et al. What influences birth place preferences, choices and decision-making amongst healthy women with straightforward pregnancies in the UK? A qualitative evidence synthesis using a 'best fit'framework approach. *BMC pregnancy and childbirth* 2017;17(1):103.
18. Coxon K, Sandall J, Fulop NJ. To what extent are women free to choose where to give birth? How discourses of risk, blame and responsibility influence birth place decisions. *Health, risk & society* 2014;16(1):51-67.
19. Scamell M. Childbirth Within the Risk Society. *Sociology Compass* 2014;8(7):917-28. doi: 10.1111/soc4.12077
20. Johanson R, Newburn M, Macfarlane A. Has the medicalisation of childbirth gone too far? *BMJ: British Medical Journal* 2002;324(7342):892.
21. MacKenzie Bryers H, van Teijlingen E. Risk, theory, social and medical models: a critical analysis of the concept of risk in maternity care. *Midwifery* 2010;26(5):488.
22. Roome S, Hartz D, Tracy S, et al. Why such differing stances? A review of position statements on home birth from professional colleges. *BJOG: An International Journal of Obstetrics & Gynaecology* 2016;123(3):376-82.
23. Ivers N, Jamtvedt G, Flottorp S, et al. Audit and feedback: effects on professional practice and healthcare outcomes. *Cochrane Db Syst Rev* 2012(6) doi: 10.1002/14651858.CD000259.pub3
24. Forsetlund L, Bjørndal A, Rashidian A, et al. Continuing education meetings and workshops: effects on professional practice and health care outcomes. *Cochrane Db Syst Rev* 2009(2) doi: 10.1002/14651858.CD003030.pub2
25. Schroeder L, Patel N, Keeler M, et al. The economic costs of intrapartum care in Tower Hamlets: A comparison between the cost of birth in a freestanding midwifery unit and hospital for women at low risk of obstetric complications. *Midwifery* 2017;45:28-35. doi: http://dx.doi.org/10.1016/j.midw.2016.11.006
26. Robinson S. Normal maternity care: whose responsibility? *BJOG: An International Journal of Obstetrics & Gynaecology* 1985;92(1):1-3. doi: doi:10.1111/j.1471-0528.1985.tb01039.x
27. Mander R, Fleming V. Failure to progress: the contraction of the midwifery profession: Psychology Press 2002.
28. Kirkham M. The culture of midwifery in the National Health Service in England. *Journal of Advanced Nursing* 1999;30(3):732-39.
29. Prosser SJ, Barnett AG, Miller YD. Factors promoting or inhibiting normal birth. *BMC Pregnancy and Childbirth* 2018;18(1):241. doi: 10.1186/s12884-018-1871-5
30. Best A, Greenhalgh T, Lewis S, et al. Large-system transformation in health care: a realist review. *The Milbank Quarterly* 2012;90(3):421-56.

For peer review only

31. Rayment J, Rance S, McCourt C, et al. Barriers to women's access to alongside midwifery units in England. *Midwifery* 2019;77:78-85. doi: https://doi.org/10.1016/j.midw.2019.06.010
32. Henshall C, Taylor B, Kenyon S. A systematic review to examine the evidence regarding discussions by midwives, with women, around their options for where to give birth. *BMC Pregnancy and Childbirth* 2016;16(1):1-13. doi: 10.1186/s12884-016-0832-0
33. McCourt C, Rayment J, Rance S, et al. Place of Birth and Concepts of Wellbeing: An Analysis from Two Ethnographic Studies of Midwifery Units in England. *Anthropology in Action* 2016;23(3):17-29.
34. Monitor. Moving healthcare closer to home: Literature review of clinical impacts. London: Monitor, 2015.
35. Hinton L, Dumelow C, Rowe R, et al. Birthplace choices: what are the information needs of women when choosing where to give birth in England? A qualitative study using online and face to face focus groups. *BMC Pregnancy and Childbirth* 2018;18(1):12. doi: 10.1186/s12884-017-1601-4
36. England N. National Maternity Review. Better Births; Improving Outcomes of Maternity Services in England. *NHS England, London* 2016
37. England N. Implementing Better Births: A resource pack for Local Maternity Systems. Publications Gateway Ref No. 06648: NHS England, 2017.

Corresponding Author

Dr Denis Walsh

Author Statement

Denis Walsh Chief Investigator, Associate Professor in Midwifery

Denis.walsh@ntlworld.com

- Principal author, drafting and revising content critically for important intellectual content, substantial contribution to the interpretation of data, final approval of the version to be published, agreement to be accountable for all aspects of the work in ensuring that questions related to the accuracy or integrity of any part of the work are appropriately investigated and resolved

Helen Spiby Co-Investigator, Professor in Midwifery

Helen.spiby@nottingham.ac.uk

- Drafting and revising content critically for important intellectual content, substantial contribution to the interpretation of data, final approval of the version to be published, agreement to be accountable for all aspects of the work in ensuring that questions related to the accuracy or integrity of any part of the work are appropriately investigated and resolved

Christine McCourt Co-Investigator, Professor in Maternal Health

Christine.mccourt.1@city.ac.uk

For peer review only

- Drafting and revising content critically for important intellectual content, substantial contribution to the interpretation of data, final approval of the version to be published, agreement to be accountable for all aspects of the work in ensuring that questions related to the accuracy or integrity of any part of the work are appropriately investigated and resolved

Celia Grigg Research Fellow

celia.grigg@xtra.co.nz

- Drafting and revising content critically for important intellectual content, substantial contribution to the interpretation of data, final approval of the version to be published, agreement to be accountable for all aspects of the work in ensuring that questions related to the accuracy or integrity of any part of the work are appropriately investigated and resolved

Dawn Coleby Early Career Academic Fellow

dawn.coleby@dmu.ac.uk

- Drafting and revising content critically for important intellectual content, substantial contribution to the interpretation of data, final approval of the version to be published, agreement to be accountable for all aspects of the work in ensuring that questions related to the accuracy or integrity of any part of the work are appropriately investigated and resolved

Simon Bishop Co-Investigator, Senior Lecturer in Business School

Simon.Bishop@nottingham.ac.uk

- Drafting and revising content critically for important intellectual content, substantial contribution to the interpretation of data, final approval of the version to be published, agreement to be accountable for all aspects of the work in ensuring that questions related to the accuracy or integrity of any part of the work are appropriately investigated and resolved

Miranda Scanlon Co-Investigator, Service User

Miranda.Scanlon@city.ac.uk

- Drafting and revising content critically for important intellectual content, substantial contribution to the interpretation of data, final approval of the version to be published, agreement to be accountable for all aspects of the work in ensuring that questions

For peer review only

related to the accuracy or integrity of any part of the work are appropriately investigated and resolved

Lorraine Culley Co-Investigator, Emeritus Professor of Social Science & Health

lac@dmu.ac.uk

- Drafting and revising content critically for important intellectual content, substantial contribution to the interpretation of data, final approval of the version to be published, agreement to be accountable for all aspects of the work in ensuring that questions related to the accuracy or integrity of any part of the work are appropriately investigated and resolved

Jane Wilkinson GP Commissioner

jane.wilkinson14@nhs.net

- Drafting and revising content critically for important intellectual content, final approval of the version to be published, substantial contribution to the interpretation of data, agreement to be accountable for all aspects of the work in ensuring that questions related to the accuracy or integrity of any part of the work are appropriately investigated and resolved

Lynne Pacanowski Head of Midwifery

homeofthepacs@gmail.com

- Drafting and revising content critically for important intellectual content, final approval of the version to be published, substantial contribution to the interpretation of data, agreement to be accountable for all aspects of the work in ensuring that questions related to the accuracy or integrity of any part of the work are appropriately investigated and resolved

Jim Thornton Professor of Obstetrics

Jim.Thornton@nottingham.ac.uk

- Drafting and revising content critically for important intellectual content, substantial contribution to the interpretation of data, final approval of the version to be published, agreement to be accountable for all aspects of the work in ensuring that questions related to the accuracy or integrity of any part of the work are appropriately investigated and resolved

Acknowledgements

For peer review only

We are extremely grateful to all who contributed to the study, including Heads of Midwifery for their support to the Stage 1 mapping phase; all who contributed during through interviews or focus groups to the Stage 2 case study phase and participants at the Stage 3 stakeholder Event.

We would also like to thank the members of the Service User Reference Panel for their invaluable contributions: Melissa Thomas, Leanne Stamp, Samantha Foulke, Joanne Whyler

We would like to thank Dr Juliet Rayment for her contribution to the media analysis of FMUs that had closed and 'Which? Birth Choice' for allowing us access to their data.

We would also like to acknowledge and thank Sheila Adamson at the University of Nottingham for all her work as the Research Secretary over 24 months.

For peer review only

Data Sharing Statement: Data are available in a public, open access repository:

https://www.journalslibrary.nihr.ac.uk/programmes/hsdr/140428/#/

Standards for Reporting Qualitative Research (SRQR)*

http://www.equator-network.org/reporting-guidelines/srqr/

Title and abstract

	Page/line no(s).
**Title** - Concise description of the nature and topic of the study Identifying the study as qualitative or indicating the approach (e.g., ethnography, grounded theory) or data collection methods (e.g., interview, focus group) is recommended	Page 1
**Abstract** - Summary of key elements of the study using the abstract format of the intended publication; typically includes background, purpose, methods, results, and conclusions	Page 4

Introduction

**Problem formulation** - Description and significance of the problem/phenomenon studied; review of relevant theory and empirical work; problem statement	Page 6,7
**Purpose or research question** - Purpose of the study and specific objectives or questions	Page 7, lines 17-19

Methods

**Qualitative approach and research paradigm** - Qualitative approach (e.g., ethnography, grounded theory, case study, phenomenology, narrative research) and guiding theory if appropriate; identifying the research paradigm (e.g., postpositivist, constructivist/ interpretivist) is also recommended; rationale**	Page 7, line 21,22
**Researcher characteristics and reflexivity** - Researchers' characteristics that may influence the research, including personal attributes, qualifications/experience, relationship with participants, assumptions, and/or presuppositions; potential or actual interaction between researchers' characteristics and the research questions, approach, methods, results, and/or transferability	
**Context** - Setting/site and salient contextual factors; rationale**	Page 7, lines 22-28
**Sampling strategy** - How and why research participants, documents, or events were selected; criteria for deciding when no further sampling was necessary (e.g., sampling saturation); rationale**	Page 7, lines 23-30
**Ethical issues pertaining to human subjects** - Documentation of approval by an appropriate ethics review board and participant consent, or explanation for lack thereof; other confidentiality and data security issues	Page 8, line 23
**Data collection methods** - Types of data collected; details of data collection procedures including (as appropriate) start and stop dates of data collection and analysis, iterative process, triangulation of sources/methods, and modification of procedures in response to evolving study findings; rationale**	Page 7, lines 23-30

For peer review only - http://bmjopen.bmj.com/site/about/guidelines.xhtml

**Data collection instruments and technologies** - Description of instruments (e.g., interview guides, questionnaires) and devices (e.g., audio recorders) used for data collection; if/how the instrument(s) changed over the course of the study	Page 7, lines 42-44
**Units of study** - Number and relevant characteristics of participants, documents, or events included in the study; level of participation (could be reported in results)	Page 7, lines 27-30
**Data processing** - Methods for processing data prior to and during analysis, including transcription, data entry, data management and security, verification of data integrity, data coding, and anonymization/de-identification of excerpts	Page 7, line 44
**Data analysis** - Process by which inferences, themes, etc., were identified and developed, including the researchers involved in data analysis; usually references a specific paradigm or approach; rationale**	Page 8, lines 1-16
**Techniques to enhance trustworthiness** - Techniques to enhance trustworthiness and credibility of data analysis (e.g., member checking, audit trail, triangulation); rationale**	

Results/findings

**Synthesis and interpretation** - Main findings (e.g., interpretations, inferences, and themes); might include development of a theory or model, or integration with prior research or theory	Pages 8-12
**Links to empirical data** - Evidence (e.g., quotes, field notes, text excerpts, photographs) to substantiate analytic findings	Pages 9-12

Discussion

**Integration with prior work, implications, transferability, and contribution(s) to the field -** Short summary of main findings; explanation of how findings and conclusions connect to, support, elaborate on, or challenge conclusions of earlier scholarship; discussion of scope of application/generalizability; identification of unique contribution(s) to scholarship in a discipline or field	Pages 12-15
**Limitations** - Trustworthiness and limitations of findings	Page 4, lines 37-48<br>Page 14, lines 16-23

Other

**Conflicts of interest** - Potential sources of influence or perceived influence on study conduct and conclusions; how these were managed	Page 5, lines 3-8
**Funding** - Sources of funding and other support; role of funders in data collection, interpretation, and reporting	Page 5, lines 1-2

*The authors created the SRQR by searching the literature to identify guidelines, reporting standards, and critical appraisal criteria for qualitative research; reviewing the reference lists of retrieved sources; and contacting experts to gain feedback. The SRQR aims to improve the transparency of all aspects of qualitative research by providing clear standards for reporting qualitative research.

For peer review only - http://bmjopen.bmj.com/site/about/guidelines.xhtml

**The rationale should briefly discuss the justification for choosing that theory, approach, method, or technique rather than other options available, the assumptions and limitations implicit in those choices, and how those choices influence study conclusions and transferability. As appropriate, the rationale for several items might be discussed together.

Reference:

O'Brien BC, Harris IB, Beckman TJ, Reed DA, Cook DA. **Standards for reporting qualitative research: a synthesis of recommendations.** *Academic Medicine*, Vol. 89, No. 9 / Sept 2014 DOI: 10.1097/ACM.0000000000000388

For peer review only

For peer review only - http://bmjopen.bmj.com/site/about/guidelines.xhtml
